# Individual differences in susceptibility to false memories for COVID-19 fake news

**DOI:** 10.1186/s41235-020-00262-1

**Published:** 2020-12-04

**Authors:** Ciara M. Greene, Gillian Murphy

**Affiliations:** 1 School of Psychology, University College Dublin, Dublin, Ireland; 2 School of Applied Psychology, University College Cork, Cork, Ireland

## Abstract

Exposure to ‘fake news’ can result in false memories, with possible consequences for downstream behaviour. Given the sharp rise in online misinformation during the coronavirus pandemic, it is important to understand the factors that influence the development of false memories. The present study measured susceptibility to false memories following exposure to fabricated news stories about the pandemic in a sample of 3746 participants. We investigated the effect of individual differences in (1) knowledge about COVID-19, (2) engagement with media or discussion about the coronavirus, (3) anxiety about COVID-19 and (4) analytical reasoning. Notably, objectively and subjectively assessed knowledge about COVID-19 were not significantly correlated. Objectively assessed knowledge was associated with fewer false memories but more true memories, suggesting a true discrimination between true and fake news. In contrast, participants who merely believed themselves to be very knowledgeable were more likely to report a memory for true stories, but showed no reduction in false memories. Similarly, individuals who reported high levels of media engagement or anxiety about COVID-19 reported an increase in true (but not false) memories. Finally, higher levels of analytical reasoning were associated with fewer memories for both true and fabricated stories, suggesting a stricter threshold for reporting a memory for any story. These data indicate that false memories can form in response to fake COVID-19 news and that susceptibility to this misinformation is affected by the individual’s knowledge about and interaction with COVID-19 information, as well as their tendency to think critically.

## Significance statement

The coronavirus pandemic has been associated with an unprecedented surge in online misinformation, to the extent that the World Health Organisation has warned of an ‘infodemic’ that may have significant behavioural consequences. Previous research has shown that exposure to ‘fake news’ can result in false memories, with possible consequences for subsequent behaviour. Susceptibility to these false memories is affected by individual differences in a range of variables. If online health misinformation is to be effectively countered, it is critical that we understand which factors will lead individuals to believe, remember or act on fake news. In this study, we identify a series of factors with proven links to misinformation susceptibility, and investigate their relationship with acceptance of COVID-19 fake news. We report that people who are more knowledgeable about COVID-19 or who score better on a test of analytical reasoning, are less prone to reporting false memories following exposure to fabricated stories. In contrast, people who simply *believe* themselves to be knowledgeable are more likely to report memories for true stories, but are not protected against false memories. People who are very anxious about COVID-19, or who engage with a lot of related content, are also more likely to report a memory for true (but not false) stories. These findings may help in the development of fake news interventions and suggest that a combination of interventions aimed at increasing public knowledge and encouraging critical evaluation of news stories may be most effective.

## Introduction

The months since the onset of the COVID-19 pandemic have seen a sharp rise in the quantity of online misinformation (Brennen et al. 2020; Kouzy et al. 2020). It has been argued that this ‘fake news’ may have severe consequences for public health and adherence to public health guidelines (e.g. O’Connor and Murphy 2020; Van Bavel et al. 2020; but see Greene and Murphy 2020). Exposure to fabricated news stories can result in false memories for the events depicted in the stories (Frenda et al. 2013; O’Connell and Greene 2017; Polage 2012). These memories are often reported in rich detail and are more likely when a false story aligns with an individual’s political or social views (Murphy et al. 2019). False memories may have behavioural consequences (see Laney and Loftus 2016 for a review); it is therefore important to understand the factors leading to their formation, so that effective interventions can be put in place.

There are a range of contextual factors that may influence an individual’s tendency to believe, remember or share a piece of misinformation. These include elements that increase the ‘truthiness’, or subjective feeling of truth about a story. Real-world news stories are typically accompanied by a photograph, that may or may not provide additional information about the content of the story. Research has shown that presenting a non-probative photograph alongside a text description can suggest the presence of multiple corroborating sources for the fabricated event, and thus increase truth ratings (Newman and Zhang 2020; Newman et al. 2012) and false memory rates (Strange et al. 2011). Similarly, multiple presentations of a story can trick participants into feeling an increased sense of familiarity for the events depicted—a phenomenon termed ‘illusory truth’ (Pennycook et al. 2018).

Individual differences in a range of variables, including age, intelligence and personality traits, may affect the propensity to form a false memory for misinformation (Lee 2004; Roediger and Geraci 2007; Zhu et al. 2010a, b). In addition, expertise or a high level of interest in a particular topic has been associated with an increased tendency to form false memories related to that topic (Baird 2003; Castel et al. 2007; Mehta et al. 2011; O’Connell and Greene 2017). Recent evidence has however suggested that objectively assessed subject-knowledge is associated with better discrimination in reported memories for true and fabricated stories, while interest or engagement with a topic increases the tendency to report a memory for any given story (Greene et al. 2020). This suggests that reports of false memories associated with knowledge of a topic may be the result of overclaiming, as participants are reluctant to admit ignorance of events pertaining to their area of interest (for similar findings on the effects of self-reported knowledge and overclaiming see Atir et al. 2015; Pennycook and Rand 2020). These findings have clear significance in the case of COVID-19 and the associated ‘infodemic’, as people are bombarded with enormous quantities of both true and fabricated health information. This deluge of information may also contribute to high rates of virus-anxiety (Jungmann and Witthöft 2020). It is not known if individuals who are particularly anxious about the public health situation, or who consider themselves to be at high risk for COVID-19, may be particularly susceptible to forming or reporting false memories for related news items.

Reporting false memories for fake news stories has been associated with lower levels of cognitive ability (Murphy et al. 2019) and analytical reasoning (Greene et al. 2020). These findings fit well with research linking critical-thinking deficits with belief in fake news (Bago et al. 2020; Pennycook and Rand 2019, 2020), and with increased acceptance of COVID-19 misinformation (Pennycook et al. 2020a, b). Notably, Martire et al. (2020), writing in the present special issue of *Cognitive Research: Principles and Implications*, report that even individuals who endorse clearly implausible claims (e.g. “the earth is flat”) are more persuaded by high-quality evidence than low quality evidence. This suggests that at least some critical analysis is taking place; the authors argue that it may be more appropriate to argue that individuals who accept the implausible claims sometimes found in fake news engage in *less* reflective thinking rather than none at all.

Understanding the role of these factors in the context of the coronavirus crisis is the key first step in developing effective interventions; for example, a focus on increasing public knowledge will be ineffective if misinformation acceptance is driven, not by ignorance, but by a failure to critically analyse unverified content.

### The present study

We investigated the effect of individual differences in a range of variables on the formation of false memories for fabricated news items. These include objectively assessed and subjectively perceived knowledge about, engagement with and anxiety relating to COVID-19, as well as individual differences in critical reasoning. We hypothesised that perceived knowledge, anxiety and engagement with COVID-related media would be associated with a bias towards reporting memories for related stories, resulting in increased rates of both true and false memories. In contrast, we expected objectively assessed knowledge about the coronavirus to be associated with better discrimination between true and fabricated stories, resulting in increased true memories but decreased false memories. We also predicted that analytical reasoning ability would be associated with a higher threshold for reporting any memory at all, and therefore reduced reports of both true and false memories.

## Methods

### Participants

Participants were recruited via an article on the Irish news website TheJournal.ie. The full sample included 4228 participants; following exclusion of those who failed attention checks (*N* = 395) or admitted looking up their answers online (*N* = 87), analysis was conducted on 3746 participants (66% female; mean age = 42.29, SD = 12.76, range = 18–101). Full details of age and education breakdown of the sample may be found in Additional file 1. The final sample provides 95% power to detect small effects (*f* < 0.1) in the regression analyses described below.

### Design and preregistration

This study was part of a larger investigation into the effects of COVID-19 fake news (preregistered at https://aspredicted.org/de7da.pdf). Participants were assigned to one of four warning conditions in a between-subjects design. Hypotheses related to the effects of warning condition and effects of fake news exposure on health behaviours are addressed in Greene and Murphy (2020). As no significant effect of warning condition was observed, data in the present paper are collapsed across groups. Hypotheses for the present paper were not explicitly preregistered, but the design and analysis plan were. We have however deviated from our preregistered plan in a number of respects:

1. In addition to an analysis of false memory rates, we also include an analysis of true memory rates, using the same predictor variables. This analysis is necessary to allow us to distinguish true false memories from a tendency towards overclaiming (i.e. by reporting memories for all stories, true and false).
2. The regression analyses include the variable of perceived knowledge, which was not listed in our preregistration. On reflection we believe it is important to compare the effects of objectively and subjectively assessed knowledge. The original preregistered analysis may be seen in Additional file 1; the inclusion of the perceived knowledge variable does not materially alter the effects of the other predictors.
3. Our preregistration called for multiple linear regression of the predictor variables on false memory count. On examination of the false memory data, it was apparent that they were better modelled using a Poisson distribution. We have therefore elected to analyse the false memory data using Poisson regression, while retaining linear regression for analysis of the true memory data.^1^ Importantly, this decision does not affect our findings; the preregistered linear regression may be seen in Additional file 1, and the pattern of results is identical.

### Materials

Participants were presented with two of the following four fabricated stories. As inclusion of photographs may increase false memory reports for fake news stories (Strange et al. 2011), each story was accompanied by a non-probative photograph (see online materials at https://osf.io/mfnb4/).

1. “New research from Harvard University shows that the chemical in chilli peppers that causes the ‘hot’ sensation in your mouth reduces the replication rate of coronaviruses. The researchers are currently investigating whether adding more spicy foods to your diet could help combat COVID-19”.
2. “A whistleblower report from a leading pharmaceutical company was leaked to the Guardian newspaper in April. The report stated that the coronavirus vaccine being developed by the company causes a high rate of complications, but that these concerns were being disregarded in favour of releasing the vaccine quickly”.
3. “A study conducted in University College London found that those who drank more than three cups of coffee per day were less likely to suffer from severe Coronavirus symptoms. Researchers said they were conducting follow-up studies to better understand the links between caffeine and the immune system”.
4. “The programming team who designed the HSE^2^ app to support coronavirus contact-tracing were found to have previously worked with Cambridge Analytica, raising concerns about citizen’s data privacy. The app is designed to monitor people’s movements in order to support the government’s contact-tracing initiative”.

Participants also viewed four true stories (see Additional file 1), concerning a study suggesting Vitamin D may protect against COVID-19, Sweden’s pandemic response, Irish politician Mary Lou McDonald cancelling political events, and MMA fighter Conor McGregor calling for stricter lockdown in Ireland.

#### COVID-19 knowledge test

A 10-item multiple choice test assessed knowledge about COVID-19 and the official response to it in Ireland. The questions concerned information that was heavily reported in news media in the weeks before the study. To ensure an appropriate level of difficulty, the test was piloted with a separate sample of 50 participants. All test items may be seen in Additional file 1.

#### Cognitive reflection test (CRT)

The CRT is a test of analytical reasoning first developed by Frederick (2005). It consists of verbal problems, each of which has an intuitive, but incorrect, solution and a correct solution that requires slower, more reasoned analysis. Several of the original CRT items are now very well known, leading to concerns that familiarity with the problem set may inflate scores. We therefore used the seven-item version of the task (Thomson and Oppenheimer 2016; Toplak et al. 2014), including reworded items which preserve the validity of the test while overcoming any familiarity effects (Manfredi and Nave 2019; Patel et al. 2019). Full details may be found in Additional file 1.

### Procedure

Data were collected online via the survey platform Qualtrics. Participants were invited to participate via an article hosted on TheJournal.ie, which included a link to the study landing page. Participants were informed that the aim of the study was to investigate reactions to public health messages and news stories relating to COVID-19. Participants provided demographic information before viewing three study blocks, presented in counterbalanced order. The first block assessed engagement with news and discussions about the coronavirus outbreak. Using a 5-point scale, participants were asked to indicate how frequently they had (1) consumed traditional media (e.g. newspaper articles, television, radio), (2) engaged with social media content and (3) had discussions with family and friends about COVID-19. They were then asked to estimate their knowledgeability about COVID-19 (1 = among the least knowledgeable people, 5 = more knowledgeable than 95% of people), and to indicate whether they had already been infected or considered themselves to be in a ‘high-risk’ category (9-point scale: 1 = definitely not, 9 = definitely). Finally, participants were asked to rate how anxious or worried they had felt since the pandemic began (1 = a lot less than usual, 9 = a lot more than usual) and how afraid they were that they or someone close to them would become ill from COVID-19 (1 = not at all afraid, 9 = very afraid).

The second block included the public health posters (described in Greene and Murphy 2020), followed by the news stories, presented in random order. After each story, participants were asked, “Do you have a memory of the events described in this story?” and selected a response from the options, “I have a clear memory of seeing/hearing about this”, “I have a vague memory of this event occurring”, “I don’t have a memory of this, but it feels familiar”, “I remember this differently” or “I don’t remember this”. Participants were asked to indicate where they had encountered the story, by selecting all applicable responses from a list (television, newspaper, radio, online news website, social media, word-of-mouth or other source) or selecting “I didn’t see/hear about this” or “I don’t remember where I saw/heard about this”. Finally, participants were asked to record how they felt about the event at the time.

The third study block comprised the 10-item multiple choice COVID-19 knowledge quiz. After completing all three blocks, participants were asked about their intentions with regard to ten health behaviours. These data are presented in Greene and Murphy (2020) and are not discussed further here. Participants were next informed that they may have been shown some fake news stories, and were asked to rate the truthfulness of the six stories they had seen (0 = definitely not true, 100 = definitely true). Finally, participants were invited to complete the CRT and debriefed (the debriefing procedure is detailed in Additional file 1). Recent evidence shows that similar debriefings are effective and eliminate persistent false memories (Murphy et al. 2020). Participation took approximately 15 min in total.

## Results

A story was deemed to be remembered if participants reported either a clear or vague memory of the events. In total, 845 participants (22.56%) reported a false memory for at least one fabricated story, while 3665 participants (97.84%) reported a memory for at least one true story. Details of the response to each story may be found in Table 1. On average, participants recalled 0.25 fabricated stories (SD = 0.49) and 2.65 true stories (SD = 1.04). Participants who reported memories for both true and fabricated stories provided more specific sources for the true stories (*M* = 1.79, SD = 0.99) than for the fabricated stories (*M* = 1.15, SD = 0.96; *t* (835) = − 17.61, *p* < 0.001, *d* = − 0.61). The fabricated stories were generally rated as less truthful than the true stories, with the exception of the contact-tracing story which was rated similarly to the four true stories (see Table 1).

**Table 1:** Responses to true and fabricated stories, including proportion of participants who endorsed each response option and mean truthfulness rating for each story

Response	Fabricated stories			
	Chilli peppers	Coffee	COVID-19 vaccine	Contact-tracing app
I have a clear memory of seeing/hearing about this	30 (1.61%)	57 (3.09%)	46 (2.49%)	193 (10.36%)
I have a vague memory of this event occurring	85 (4.56%)	138 (7.49%)	125 (6.77%)	282 (15.14%)
I don’t have a memory of this, but it feels familiar	88 (4.73%)	114 (6.19%)	182 (9.85%)	172 (9.23%)
I remember this differently	17 (0.91%)	12 (0.65%)	29 (1.57%)	93 (4.99%)
I don't remember this	1642 (88.18%)	1522 (82.58%)	1465 (79.32%)	1123 (60.28%)
Average truthfulness rating [mean (SD)]	15.62 (21.54)	19.20 (24.50)	26.79 (28.09)	60.24 (34.97)
Total *N*	1862	1843	1847	1863

	True stories			
	Vitamin D	Conor McGregor	Mary Lou McDonald	Sweden
I have a clear memory of seeing/hearing about this	1295 (34.83%)	943 (25.17%)	1701 (45.85%)	2836 (76.61%)
I have a vague memory of this event occurring	888 (23.88%)	908 (24.24%)	823 (22.18%)	540 (14.59%)
I don’t have a memory of this, but it feels familiar	369 (9.92%)	290 (7.83%)	252 (6.79%)	115 (3.11%)
I remember this differently	319 (8.58%)	172 (4.65%)	270 (7.28%)	103 (2.78%)
I don't remember this	847 (22.61%)	1390 (37.54%)	664 (17.90%)	108 (2.92%)
Average truthfulness rating [mean (SD)]	60.94 (34.31)	51.03 (38.68)	70.77 (32.60)	87.49 (20.55)
Total *N*	3718	3703	3710	3702

### Predictors of false memories for COVID-19 misinformation

Multiple regressions were conducted for true and false memory count, with the predictor variables (1) COVID-19 knowledge (*M* = 6.82, SD = 1.29), (2) perceived knowledge (*M* = 3.37, SD = 0.82), (3) CRT score (*M* = 3.52, SD = 1.91), (4) engagement with COVID-related media and discussions, and (5) anxiety about COVID-19. Engagement was defined as the average of three items assessing engagement with traditional media, social media and discussions with family and friends; participants typically reported being very engaged with this topic (*M* = 4.04, SD = 0.74). COVID-19 anxiety was defined as the average of three items assessing general anxiety, fear of contracting COVID-19 and the participants’ judgement of their own high-risk status (*M* = 5.52, SD = 1.67). The true memory count data were analysed using linear regression, while Poisson regression was used to analyse the false memory count data. Small to moderate correlations were observed among the predictor variables (0.01 ≥ *r* ≤ 0.15); the zero-order correlations and multicollinearity diagnostics may be found in Additional file 1. Notably, objectively and subjectively assessed knowledge was not significantly correlated (*r* = 0.03).

**Fig. 1:**
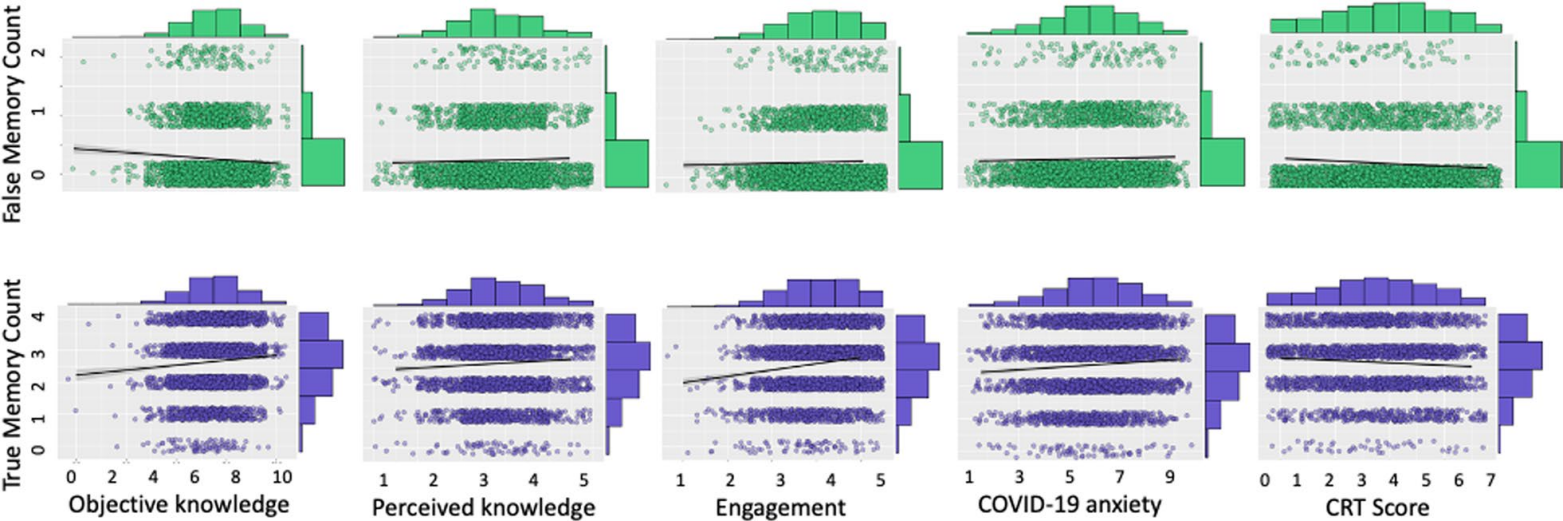
Scatterplot matrix with jittered datapoints and marginal distributions, depicting the relationship of false memory count (top row) and true memory count (bottom row) with each of the predictor variables

Both models were found to significantly predict the outcome variable (false memory count: *χ*^2^ (5) = 38.67, *N* = 3053, *p* < 0.001; true memory count: *R*^2^ = 0.04, *F*(5,3047) = 24.22, *p* < 0.001). The coefficients of regression are reported in Table 2, and the relationships among variables are depicted in Fig. 1. Greater objectively assessed knowledge about COVID-19 was associated with a reduced tendency to form false memories following exposure to fake news, and an increased tendency to report true memories. In contrast, perceived knowledgeability was associated with an increased tendency to remember true stories but had no effect on false memories. Participants who engaged more with COVID-related media reported more true memories but did not respond differently to false stories. Similarly, anxiety about COVID-19 was associated with increased memory reports for true stories but not false stories. Finally, this analysis revealed that analytical reasoning was associated with a reduction in reports of both true and false memories, suggesting that participants with higher CRT scores were less likely to report a memory for any story, regardless of its truth value.

**Table 2:** Regression coefficients for individual predictors of susceptibility to COVID-19 misinformation

Predictor	*B*	SE (*B*)	*β*^a^	Wald *χ*^2^	*p*	95% CI (*B*)	
						Lower	Upper
*False memory count*							
(Intercept)	− 1.136	0.32	0.32	12.90	< 0.001	− 1.76	− 0.52
Objective knowledge*	− 0.10	0.03	0.91	11.71	< 0.001	− 0.15	− 0.04
Perceived knowledge	0.07	0.05	1.07	2.42	0.12	− 0.02	0.16
Engagement	0.07	0.05	1.07	1.88	0.17	− 0.03	0.17
COVID-19 anxiety	0.02	0.02	1.02	1.03	0.31	− 0.02	0.07
CRT score*	− 0.08	0.02	0.92	16.61	< 0.001	− 0.12	− 0.04

	*B*	SE (*B*)	*β*^a^	*t*	*p*	95% CI (*B*)	
						Lower	Upper
*True memory count*							
(Intercept)	1.30	0.16		8.34	< 0.001	0.99	1.61
Objective knowledge*	0.05	0.01	0.06	3.57	< 0.001	0.03	0.08
Perceived knowledge*	0.07	0.02	0.06	3.14	0.002	0.03	0.11
Engagement*	0.17	0.025	0.125	6.88	< 0.001	0.12	0.22
COVID-19 anxiety*	0.04	0.01	0.065	3.62	< 0.001	0.02	0.06
CRT score*	− 0.04	0.01	− 0.07	− 3.84	< 0.001	− 0.05	− 0.02

* *p* < 0.05

^a^For Poisson regressions (i.e. false memory count), β (Exp (*B*)) is given as 1 for no effect, with values > 1 for positive effects and < 1 for negative effects. For linear regressions (i.e. true memory count), *β* is given as 0 for no effect with values < 0 for negative effects and > 0 for positive effects

## Discussion

The aim of this study was to investigate individual differences in susceptibility to false memories for COVID-19 misinformation. In contrast with past evidence suggesting that greater subject-knowledge or expertise may increase false memories (Baird 2003; Castel et al. 2007; Mehta et al. 2011; O’Connell and Greene 2017), we report that objectively assessed knowledge about COVID-19 was associated with *fewer* false memories, but more true memories. This suggests that actual, measurable knowledge of a topic enhances the ability to discriminate between true and false stories, and reduces the tendency to construct false memories when faced with novel material. In contrast, participants who *believed* themselves to be more knowledgeable were more likely to report a memory for true stories, but were not less likely to report false memories—in fact, a small, non-significant trend towards increased false memories was observed. This is in line with the hypothesis that perceived knowledge may lead to a tendency towards overclaiming, if participants are unwilling to admit to not remembering a given event. Participants who reported high levels of engagement with or anxiety about COVID-19 were also likely to report more true memories, with no effect on false memories. Engagement with and anxiety about COVID-19 were only moderately correlated (*r* = 0.15), but their effects on false memory formation were similar (though engagement was the stronger predictor). These findings align with those reported by Greene et al. (2020), and provide converging support for the idea that true knowledge about a topic and self-reported knowledge or engagement with that topic exert independent effects on memory.

In line with previous research (Greene et al. 2020), higher levels of analytical reasoning were associated with fewer false memories, and also with fewer true memories. This suggests a response bias, such that less analytical participants showed a stronger bias towards reporting a memory. Thus, the decrease in false memories can be attributed to a higher reporting threshold (i.e. a greater degree of suspicion about the stories in general) among more analytical respondents. Individuals with stronger critical thinking skills may require further evidence before reporting a memory for a story that sounds vaguely familiar; this finding therefore underlines the importance of critical thought in reducing the impact of misinformation (Martire et al. 2020; Pennycook et al. 2020; Pennycook and Rand 2018, 2019).

There are some limitations that should be considered when interpreting these data. First, the nature of the experiment means that we cannot rule out the possibility that our methods uncovered existing false memories about the events described in the fabricated stories, rather than implanting them. We consider it unlikely that participants would have spontaneously generated false memories about the highly specific fictional events described in the stories, but nevertheless we must acknowledge this as a possibility. Second, many of the effect sizes reported in Table 2 are relatively small, suggesting that some of the variables assessed may have little impact on real-world behaviour.

In summary, these data demonstrate that false memories can occur following exposure to fake news about COVID-19, albeit at a lower rate than those reported elsewhere (Greene et al. 2020; Murphy et al. 2019). False memories were especially likely among individuals who are less analytical and less knowledgeable about the pandemic; in contrast, individuals who believed themselves to be very knowledgeable, or who reported high virus-related anxiety and frequent engagement with related media, reported more true memories but did not report correspondingly fewer false memories. Objectively and subjectively-assessed knowledge were not significantly correlated; thus, this finding cannot be explained in terms of increased information about the true stories. Instead, this suggests that self-reported involvement for a topic increases participants’ bias towards ‘remembering’ events, but does not protect against false memories. The current study suggests that interventions aimed at increasing critical thinking (e.g. Pennycook et al. 2020) or improving subject-knowledge, may help to reduce susceptibility to COVID-19-related fake news. In addition, governments may wish to consider proposals to include media literacy and critical thinking training in school curricula (e.g. Frechette 2019), to better prepare the next generation to discriminate true news from false.

## Supplementary Information

**Additional file 1**. Supplementary materials and analysis for “Individual differences in susceptibility to false memories for COVID-19 fake news”.

## Data Availability

The study materials and data are available in the Open Science Framework repository, at https://osf.io/mfnb4/.

## Footnotes

1 The true memory count data could have been modelled using either a normal or Poisson distribution, but more closely met the assumptions of linear regression.

2 Health Service Executive, Ireland’s public health service provider.
