# Supplementary material for: Individual differences in susceptibility to false memories for COVID-19 fake news
Source: Cogn Res Princ Implic. 2020 Dec 4;5:63. doi: 10.1186/s41235-020-00262-1 (PMC7716111; doi:10.1186/s41235-020-00262-1)
Supplement: Supplementary file 1 — Additional file 1. Supplementary materials and analysis for “Individual differences in susceptibility to false memories for COVID-19 fake news”. [file 41235_2020_262_MOESM1_ESM.docx]

**Additional File 1**

**Supplementary materials and analysis for “Individual differences in susceptibility to false memories for COVID-19 fake news”**

***True stories***

All participants viewed the following four true stories, which were based on real events described in Irish media in the weeks prior to the study. Each story was accompanied by a non-probative photograph.

1. A new study from Trinity College Dublin revealed that vitamin D is likely to reduce serious coronavirus complications. The researchers urged the government to advise Irish citizens to take daily vitamin D supplements. [Accompanying photograph: a bottle of unlabelled pills, beside an image of a woman wearing a facemask.]
2. Mixed-martial arts fighter Conor McGregor posted an online video urging the Irish government to enforce a complete lockdown, with the help of the army. “I urge our government to utilise our defence forces” he stated. [Accompanying photograph: A video still of Mr. McGregor speaking to camera].
3. Sinn Féin President Mary Lou McDonald called off two Sinn Féin rallies in March, after a case of coronavirus was reported at her children's school. [Accompanying photograph: a posed photograph of Ms. McDonald].
4. As most of Europe is in lockdown, Sweden is pursuing a different strategy against COVID-19. Pubs, restaurants, gyms and most schools remain open in the Scandinavian state, with the government relying on personal responsibility for compliance rather than strict enforcement. Official guidance states that citizens may socialise, as long as they stay at “arm’s length” from each other [Accompanying photograph: people walking down a busy city street].

***COVID-19 knowledge test***

Items in this test were derived from news reports in the weeks prior to the study start date. Pilot testing with 50 participants indicated a mean score of 5.8/10 (SD = 1.96, range = 3-9).

1. What is the most effective means for preventing infection by the coronavirus?

- **Frequent hand-washing**
- Wearing a face mask
- A highly acidic diet
- Staying within 2km of your home

1. Which of the following are the main symptoms of COVID-19, according to the HSE?

- **Fever, cough, shortness of breath**
- Fever, sneezing, cough
- Fever, sinus pain, chesty cough
- Fever, fatigue, dry skin

1. Which household animals have been found to be transmit COVID-19 to owners?
   - Dogs
   - Cats
   - Snakes, lizards and other exotic reptiles
   - **None of the above**
2. When did China announce the first death from the novel coronavirus?
   - December 30^th^ 2019
   - January 1^st^ 2020
   - **January 11^th^ 2020**
   - January 26^th^ 2020
3. Which was the first country in Europe to report a confirmed case of COVID-19?
   - Italy
   - **France**
   - United Kingdom
   - Serbia
4. When did Irish schools and colleges close in response to the public health crisis?

- March 7^th^
- **March 13^th^**
- March 17^th^
- March 28^th^

1. If you are in contact with a confirmed case of COVID-19, how long should you self-isolate to avoid infecting others?

- Five days
- Seven days
- Ten days
- **Fourteen days**

1. If someone is ‘cocooning’, what restrictions are there on their movements?

- They are recommended not to leave their home
- **They are recommended not to leave their home or garden**
- They cannot go beyond 2km of their home
- They cannot leave their home except for essential groceries or medicines

1. What is the R_0_ (‘r-naught’) of a disease?
   - **The number of people an average person with the virus will infect**
   - The average number of days an infected person will experience symptoms
   - The quantity of virus to which one must be exposed to become sick
   - The ratio of exposed individuals who become sick to those who do not
2. In which order will the following services and businesses reopen in Ireland?
   - **Public libraries, playgrounds, hairdressers, gyms**
   - Playgrounds, gyms, public libraries, hairdressers
   - Playgrounds, public libraries, gyms, hairdressers
   - Public libraries, gyms, hairdressers, playgrounds

***Cognitive Reflection Test (CRT)***

The original CRT (Frederick, 2005) included three numerical problems (e.g. “a bat and ball cost $1.10. If the bat costs $1 more than the ball, how much does the ball cost?”). A seven-item version of the task was later developed which includes four additional non-numerical problems (Thomson & Oppenheimer, 2016; Toplak et al., 2014). Although the predictive ability of the CRT has been found to be robust to multiple exposures (Bialek & Pennycook, 2018; Meyer et al., 2018), the three original problems are now very well-known; we therefore employed reworded versions of these three questions, (obtained from Patel et al., 2019), which preserve the structure of the problems while altering their surface content; see items 1-3 below. Rewording the CRT problems in this manner has been shown to maintain the validity of the test while overcoming any familiarity effects (Manfredi & Nave, 2019).

Reworded 7-item CRT problems:

1. A cheese and crackers snack costs €2.20 in total. The cheese costs €2.00 more than the crackers. How much do the crackers cost?

- **Correct answer: 10 cents** [Accept any variation on this; 10c, 10 c, 0.10 etc.)

1. If it takes 6 hackers 6 minutes to crack 6 passwords, how long would it take 90 hackers to crack 90 passwords?
   - **Correct answer: 6 minutes**
2. In a field, there is a patch of weeds. Every day, the patch doubles in size. If it takes 50 days for the patch to cover the entire field, how long would it take for the patch to cover half the field?
   - **Correct answer: 49 days**
3. If you’re running a race and you pass the person in second place, what place are you in?
   - **Correct answer: second place**
4. A farmer had 15 sheep and all but 8 died. How many are left?
   - **Correct answer: 8**
5. Emily’s father has three daughters. The first two are named April and May. What is the third daughter’s name? [intuitive answer: June; correct answer: Emily]
   - **Correct answer: Emily**
6. How many cubic feet of dirt are there in a hole that is 3’ deep x 3’ wide x 3’ long?
   - **Correct answer: none (it’s a hole)**

***Demographic details***

Distribution of age and education across the final sample (N = 3746)

Age: Mean = 46.29 years, SD = 12.76, range = 18-101


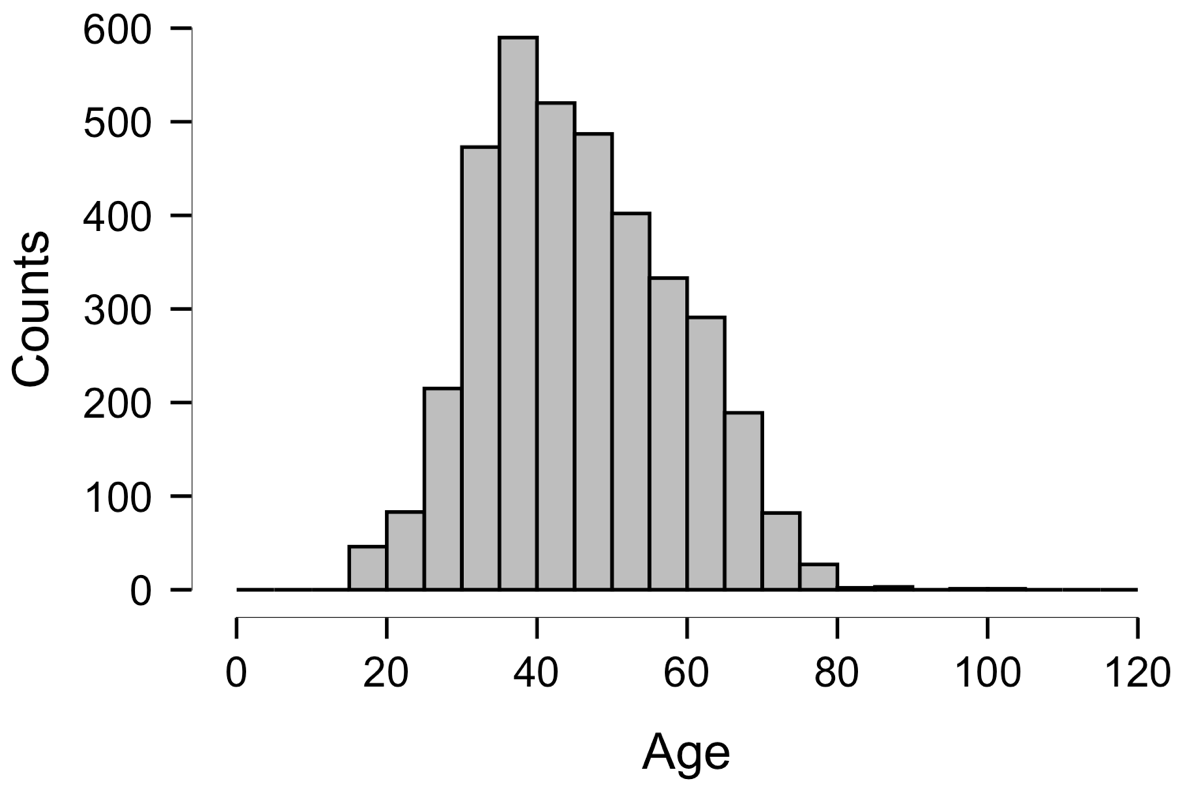


Education (see category labels below)


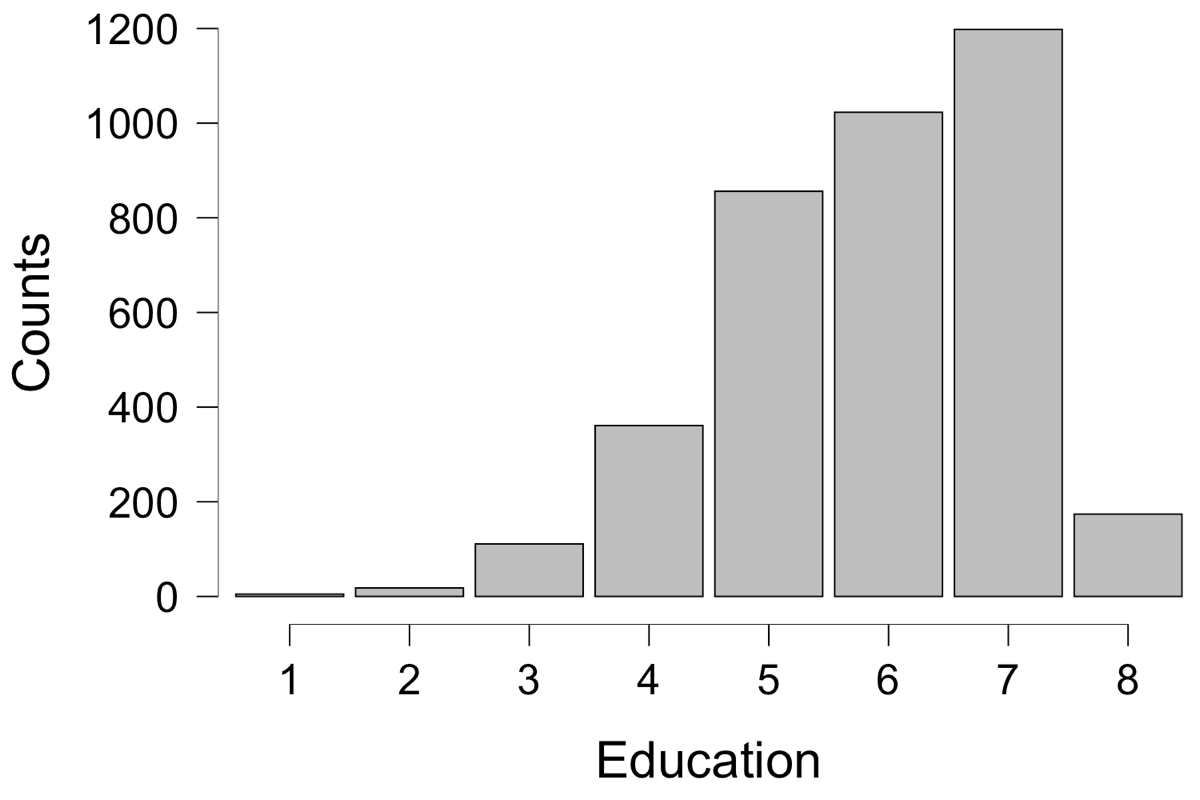


Education categories:

1. No formal education
2. Completed primary school (8 years formal education)
3. Completed Junior/Intermediate Certificate or equivalent (11 years formal education)
4. Completed Leaving Certificate (14 years formal education)
5. Some college/university
6. Completed an undergraduate degree
7. Completed a postgraduate degree
8. Completed a doctoral degree

***Zero-order correlations for regression predictors (Pearson’s r)***

No problematic multicollinearity was detected in the regression (variance inflation factor < 1.1, tolerance > 0.95 for all predictors).

|  | **Objective knowledge** | **Perceived knowledge** | **Engagement** | **COVID anxiety** | **CRT score** |
| --- | --- | --- | --- | --- | --- |
| **Objective knowledge** | — |  |  |  |  |
| **Perceived knowledge** | 0.03 | — |  |  |  |
| **Engagement** | 0.14** | 0.08** | — |  |  |
| **COVID anxiety** | -0.005 | 0.004 | 0.15** | — |  |
| **CRT score** | 0.13** | 0.01 | -0.05* | -0.10** | — |

* p < .05

** p < .001

***Preregistered linear regression analysis of false memory count***

This analysis does not include the perceived knowledge variable. The model was significant: R^2^ = 0.012, F(4, 3054) = 9.42, *p* < .001. Regression coefficients may be found in the table below.

|  |  |  |  |  |  |  | **95% CI (B)** | |
| --- | --- | --- | --- | --- | --- | --- | --- | --- |
|  | **Predictor** | **B** | **SE (B)** | **β** | ***t*** | ***p*** | **Lower** | **Upper** |
| **False memory count** | |  |  |  |  |  |  |  |
|  | (Intercept) | 0.38 | 0.07 |  | 5.38 | < .001 | 0.24 | 0.52 |
|  | Objective knowledge* | -0.02 | 0.07 | -0.06 | -3.50 | < .001 | -0.04 | -0.01 |
|  | Engagement | 0.02 | 0.01 | 0.03 | 1.46 | 0.15 | -0.01 | 0.04 |
|  | COVID-19 anxiety | 0.005 | 0.005 | 0.02 | 1.00 | 0.32 | -0.005 | 0.02 |
|  | CRT score* | -0.02 | 0.05 | -0.07 | -4.12 | < .001 | -0.03 | -0.01 |
|  | **p* < .05 |  |  |  |  |  |  |  |

***Details of debriefing procedure***

The debriefing was a multi-step process. First, participants were told ‘You were shown two entirely fabricated events - the stories below were invented by the researchers and never happened. These events are **entirely false** and should in no way be taken into account when making decisions about your health’. Each fabricated story was then re-presented along with a ‘FALSE’ label and a brief explanation (e.g. ‘This story is not true. There is no known association between the HSE contact tracing app and Cambridge Analytica’). Participants who chose to do so then completed the CRT before being directed to a fully debriefing. Participants who chose not to compete the CRT were directed straight to the debriefing. Throughout the experiment, participants who wished to halt their participation were urged to click a prominent button labelled ‘End Study Now’ which redirected them to the debriefing, rather than simply closing their browser.

The debriefing consisted of a detailed explanation of the study aims, along with links to reliable sources for information about COVID-19 and advice for protecting one’s mental health during the pandemic (e.g. the World Health Organisation and the Health Service Executive of Ireland). Participants were asked to reconfirm that they were happy for their data to be used in the study. Finally, participants were directed to a series of questions about the debriefing, in which they rated a series of statements including “I am confident in my understanding of which stories were true and which were fabricated” and “I feel I learned something by participating in this study (e.g. about fake news or false memories)”.
